# Supplementary material for: Probiotic Supplementation during the Perinatal and Infant Period: Effects on gut Dysbiosis and Disease
Source: Nutrients. 2020 Jul 27;12(8):2243. doi: 10.3390/nu12082243 (PMC7468726; doi:10.3390/nu12082243)
Supplement: Supplementary file 1 [file nutrients-12-02243-s001.pdf]

Supplementary Materials

Search methodology

Section 3.1. MeSH terms (PubMed) and search: "pregnancy"[MeSH Terms] OR pregnancy [Text Word], "microbiota"[MeSH Terms] OR "microbiome"[Text Word], "colonization"[All Fields], "growth and development"[Subheading] OR "development" [Text Word]. Filters: Article types: case reports, classical article, clinical study, clinical trial, comparative study, controlled clinical trial, multicenter study, observational study, randomized controlled trial; Publication dates: 2004–2019 (inclusive); Species: humans; Language: English; Age: infants.

Section 3.2. No MeSH terms (PubMed) and search: "Microbiota"[All Fields], AND "Intrapartum antibiotic prophylaxis"[All Fields] OR "neonatal antibiotic therapy"[All Fields]; "Dysbiosis"[All Fields], AND "neonatal antibiotic therapy"[All Fields] OR "Intrapartum antibiotic prophylaxis"[All Fields]. Filters: Article types: case reports, classical article, clinical study, clinical trial, comparative study, controlled clinical trial, multicenter study, observational study, randomized controlled trial; Publication dates: 2004–2019 (inclusive); Species: humans; Language: English; Age: infants.

Section 3.3. MeSH terms (PubMed) and search: (((("dysbiosis"[MeSH Terms] OR "dysbiosis"[All Fields]) OR "dysbioses"[All Fields]) AND (((("microbiota"[MeSH Terms] OR "microbiota"[All Fields]) OR "microbiotas"[All Fields]) OR "microbiota s"[All Fields]) OR "microbiotae"[All Fields])) AND (((((((((("allergie"[All Fields] OR "hypersensitivity"[MeSH Terms]) OR "hypersensitivity"[All Fields]) OR "allergies"[All Fields]) OR "allergy"[All Fields]) OR "allergy and immunology"[MeSH Terms]) OR ("allergy"[All Fields] AND "immunology"[All Fields])) OR "allergy and immunology"[All Fields]) OR (((("disease"[MeSH Terms] OR "disease"[All Fields]) OR "diseases"[All Fields]) OR "disease s"[All Fields]) OR "diseased"[All Fields])) OR (((("food hypersensitivity"[MeSH Terms] OR ("food"[All Fields] AND "hypersensitivity"[All Fields])) OR "food hypersensitivity"[All Fields]) OR ("food"[All Fields] AND "allergy"[All Fields])) OR "food allergy"[All Fields])) OR ((("dermatitis, atopic"[MeSH Terms] OR ("dermatitis"[All Fields] AND "atopic"[All Fields])) OR "atopic dermatitis"[All Fields]) OR ("atopic"[All Fields] AND "dermatitis"[All Fields]))) OR ((("asthma"[MeSH Terms] OR "asthma"[All Fields]) OR "asthmas"[All Fields]) OR "asthma s"[All Fields]))) AND ("bifidobacterium"[MeSH Terms] OR "bifidobacterium"[All Fields]). Filters: Article types: Full text, Clinical Trial, Meta-Analysis, Randomized Controlled Trial, Review, Systematic Reviews; Publication dates: 2004–2020 (inclusive); Species: humans; Language: English; Ages: Children: birth–18 years, Newborns: birth–1 month, Infants: birth–23 months, Preschool Children: 2–5 years, Children: 6–12 years.

Section 3.4. MeSH terms (PubMed) and search: (((((("probiotic s"[All Fields] OR "probiotical"[All Fields]) OR "probiotics"[MeSH Terms]) OR "probiotics"[All Fields]) OR "probiotic"[All Fields]) OR ("bifidobacterium"[MeSH Terms] OR "bifidobacterium"[All Fields])) AND ((((("milk, human"[MeSH Terms] OR ("milk"[All Fields] AND "human"[All Fields])) OR "human milk"[All Fields]) OR ("human"[All Fields] AND "milk"[All Fields])) OR (((((("breast feeding"[MeSH Terms] OR ("breast"[All Fields] AND "feeding"[All Fields])) OR "breast feeding"[All Fields]) OR "breastfeeding"[All Fields]) OR "breastfeedings"[All Fields]) OR "breastfeeders"[All Fields]))) OR (((("milk, human"[MeSH Terms] OR ("milk"[All Fields] AND "human"[All Fields])) OR "human milk"[All Fields]) OR ("breast"[All Fields] AND "milk"[All Fields])) OR "breast milk"[All Fields]))) AND (((((((("transfer"[All Fields] OR "transferability"[All Fields]) OR "transferable"[All Fields]) OR "transfered"[All Fields]) OR "transfering"[All Fields]) OR "transferred"[All Fields]) OR "transferring"[All Fields]) OR "transfers"[All Fields]) OR ((((((((((((((("translocalization"[All Fields] OR "translocalized"[All Fields]) OR "translocants"[All Fields]) OR "translocate"[All Fields]) OR "translocated"[All Fields]) OR "translocates"[All Fields])

OR "translocating"[All Fields]) OR "translocation, genetic"[MeSH Terms]) OR ("translocation"[All Fields] AND "genetic"[All Fields])) OR "genetic translocation"[All Fields]) OR "translocation"[All Fields]) OR "protein transport"[MeSH Terms]) OR ("protein"[All Fields] AND "transport"[All Fields])) OR "protein transport"[All Fields]) OR "translocations"[All Fields]) OR "translocational"[All Fields]) OR "translocator"[All Fields]) OR "translocators"[All Fields])). Filters: Article type: Full text, Clinical Trial, Meta-Analysis, Randomized Controlled Trial, Review, Systematic Reviews; Publication dates: 2004–2020 (inclusive); Species: humans; Language: English; Ages: Children: birth–18 years, Newborns: birth–1 month, Infants: birth–23 months, Preschool Children: 2–5 years, Children: 6–12 years..

Section 3.5. MeSH terms (PubMed) and search: ((((("probiotic s"[All Fields] OR "probiotical"[All Fields]) OR "probiotics"[MeSH Terms]) OR "probiotics"[All Fields]) OR "probiotic"[All Fields]) AND ((((("food hypersensitivity"[MeSH Terms] OR ("food"[All Fields] AND "hypersensitivity"[All Fields])) OR "food hypersensitivity"[All Fields]) OR ("food"[All Fields] AND "allergy"[All Fields])) OR "food allergy"[All Fields]) OR (("food hypersensitivity"[MeSH Terms] OR ("food"[All Fields] AND "hypersensitivity"[All Fields])) OR "food hypersensitivity"[All Fields]))) AND (("lactobacillus"[MeSH Terms] OR "lactobacillus"[All Fields]) OR ("bifidobacterium"[MeSH Terms] OR "bifidobacterium"[All Fields]))**.** Filters: Article types: Full-text, Randomized Controlled Trial, Publication dates: 2004–2019 (inclusive); Species: humans; Language: English; Ages: infants (birth–23 months and newborns: birth–1 month).

Section 3.6. MeSH terms (PubMed) and search: ((((("probiotic s"[All Fields] OR "probiotical"[All Fields]) OR "probiotics"[MeSH Terms]) OR "probiotics"[All Fields]) OR "probiotic"[All Fields]) AND ((((("asthma"[MeSH Terms] OR "asthma"[All Fields]) OR "asthmas"[All Fields]) OR "asthma s"[All Fields]) OR (((((("respiratory sounds"[MeSH Terms] OR ("respiratory"[All Fields] AND "sounds"[All Fields])) OR "respiratory sounds"[All Fields]) OR "wheeze"[All Fields]) OR "wheezes"[All Fields]) OR "wheezing"[All Fields]) OR "wheezed"[All Fields])) OR (("rhinitis"[MeSH Terms] OR "rhinitis"[All Fields]) OR "rhinitides"[All Fields]))) AND (((((((((((((((("prevent"[All Fields] OR "preventability"[All Fields]) OR "preventable"[All Fields]) OR "preventative"[All Fields]) OR "preventatively"[All Fields]) OR "preventatives"[All Fields]) OR "prevented"[All Fields]) OR "preventing"[All Fields]) OR "prevention and control"[MeSH Subheading]) OR ("prevention"[All Fields] AND "control"[All Fields])) OR "prevention and control"[All Fields]) OR "prevention"[All Fields]) OR "prevention s"[All Fields]) OR "preventions"[All Fields]) OR "preventive"[All Fields]) OR "preventively"[All Fields]) OR "preventives"[All Fields]) OR "prevents"[All Fields]). Filters: Article types: Full-text, Randomized Controlled Trial, Publication dates: 2009–2019 (inclusive); Species: humans; Language: English.

Section 3.7. MeSH terms (PubMed) and search: "Probiotics"[MeSH Terms] OR "para-probiotics"[Text Word], AND "preterm infant"[MeSH Terms] OR "preterm neonate"[MeSH Terms]. Filters: Article type: case reports, classical article, clinical study, clinical trial, comparative study, controlled clinical trial, journal article, multicenter study, observational study, randomized controlled trial); Publication dates: 2004–2019 (inclusive); Species: humans; Languages: English; Ages: infants (birth–23 months and newborns: birth–1 month).

Section 3.8. MeSH terms (Pubmed) and search: (("Probiotics"[Majr] AND "Safety"[Mesh]) AND (("Pregnancy"[Mesh:NoExp]) OR "Infant, Newborn"[Mesh])) OR ((("Probiotics"[Majr] AND "Safety"[Mesh]) AND (("Pregnancy"[Mesh:NoExp]) OR "Infant, Newborn"[Mesh]))[All Fields] NOT medline[sb]). Filters: Article type: case reports, classical article, clinical study, clinical trial, comparative study, controlled clinical trial, journal article, multicenter study, observational study,

randomized controlled trial); Publication dates: 2009–2019 (inclusive); Species: humans; Languages: English, Spanish; Ages: adults (19–44 years) and newborns (birth–1 month)
